# Association of childhood and adult socioeconomic indicators with cardiovascular risk factors and its modification by age: the CARLA Study 2002-2006

**DOI:** 10.1186/1471-2458-11-289

**Published:** 2011-05-10

**Authors:** Barbara Schumann, Alexander Kluttig, Daniel Tiller, Karl Werdan, Johannes Haerting, Karin H Greiser

**Affiliations:** 1 Institute of Medical Epidemiology, Biostatistics, and Informatics, Martin-Luther-University Halle-Wittenberg, 06097 Halle (Saale), Germany; 2 Department of Public Health and Clinical Medicine, Epidemiology and Global Health, Umeå University, 901 87 Umeå, Sweden; 3 Department of Medicine III, Martin-Luther-University Halle-Wittenberg, 06097 Halle (Saale), Germany; 4 German Cancer Research Centre, Division of Cancer Epidemiology, 69120 Heidelberg, Germany

## Abstract

**Background:**

The influence of socioeconomic status (SES) on cardiovascular diseases and risk factors is widely known, although the role of different SES indicators is not fully understood. The aim of this study was to investigate the role of different SES indicators for cardiovascular disease risk factors in a middle and old aged East German population.

**Methods:**

Cross-sectional data of an East German population-based cohort study (1779 men and women aged 45 to 83) were used to assess the association of childhood and adulthood SES indicators (childhood SES, education, occupational position, income) with cardiovascular risk factors. Adjusted means and odds ratios of risk factors by SES indicators with 95% confidence intervals (CI) were calculated by linear and logistic regression models, stratified by sex. The interaction effect of education and age on cardiovascular risk factors was tested by including an interaction term.

**Results:**

In age-adjusted models, education, occupational position, and income were statistically significantly associated with abdominal obesity in men, and with smoking in both sexes. Men with low education had a more than threefold risk of being a smoker (OR 3.44, CI 1.58-7.51). Low childhood SES was associated with higher systolic blood pressure and abdominal obesity in women (OR 2.27, CI 1.18-4.38 for obesity); a non-significant but (in terms of effect size) relevant association of childhood SES with smoking was observed in men. In women, age was an effect modifier for education in the risk of obesity and smoking.

**Conclusions:**

We found considerable differences in cardiovascular risk factors by education, occupational position, income, and partly by childhood social status, differing by sex. Some social inequalities levelled off in higher age. Longitudinal studies are needed to differentiate between age and birth cohort effects.

## Background

The role of socioeconomic status (SES) in cardiovascular disease (CVD) and other health outcomes has been recognized for a long time [1,2]. Numerous studies show that part of the social gradient in health is explained by adverse risk factor profiles in lower socioeconomic status groups. Lifestyle-related factors such as smoking, physical inactivity, and overweight are known to be particularly related to socioeconomic factors [3-5].

Despite the richness of evidence, there are also studies showing no effect, or gender-specific differences [6-8]. Most research is conducted with one single SES indicator only. Each SES indicator, however, reflects different aspects of social stratification which might be more or less relevant to different health outcomes or health behaviours: Education measures cultural achievement, knowledge and cognitive functioning which make the individual more receptive to health education measures [9]. Thus education may be more directly related to health behaviour such as smoking than for example income. Occupational position is related to prestige, influence at the workplace and exposure to occupational hazards and other working conditions (e.g. psychosocial stress and lead exposure may cause high blood pressure, physical inactivity is associated with obesity). Income describes economic welfare and, consequently, access to resources including place of residence, recreation, food and exercise. Restricted access to such resources might contribute to an adverse risk profile [9]. SES indicators such as education, occupational position and income are only interrelated to a certain extent. Moreover, they are considered to describe SES achieved at different stages of the life course: education is achieved during early adulthood, whereas income and occupational position describe SES during later adulthood. Thus, in order to identify the most vulnerable groups and to develop instruments for cardiovascular disease prevention, differentiation of the respective components of social status is necessary. Furthermore, the role of social origin (childhood socioeconomic status) for adult health behaviour and cardiovascular risk needs to be considered in the context of adult socioeconomic status.

The influence of education on cardiovascular risk factors might vary over time for several reasons. In terms of an age effect, inequalities may increase in older age through cumulative disadvantages, or diminish during lifetime owing either to age functioning as a leveller [10], or to selective survival of individuals with higher SES [9]. On the other hand, as regards a birth cohort effect, the association between education and health risks could increase in younger age-cohorts compared with older cohorts because of changing societal appraisal of educational attainments. The meaning of educational achievements changes over time, i.e. across birth cohorts, owing to generally higher educational attainments and a secular increase in educational qualification in later birth cohorts. In Germany, the relative proportion of middle and higher educational achievements has substantially increased since World War II, leaving those of lower education with fewer occupational and life chances than older generations with the same education [11,12]. Whether these changes are indeed reflected by differing risk factors is not clearly understood.

Among the most relevant and highly prevalent cardiovascular risk factors are high blood pressure, obesity and smoking [13-16]. The aim of this study was to investigate the influence of different indicators of socioeconomic status (childhood SES, education, occupational position and income) on these cardiovascular risk factors in an ageing East German population. We further aimed to analyse the interaction of age and education on the risk of blood pressure, obesity and smoking, hypothesizing that social differences in these factors would decrease with age.

## Methods

### Study design and participants

Data of the baseline examination of the CARLA Study (Cardiovascular Disease, Living and Ageing in Halle) were used for the present analysis. The CARLA Study is a prospective cohort study with a population-based random sample of the inhabitants of Halle, a medium-sized city in Eastern Germany. The study population comprised 1779 participants aged 45 to 83 at baseline (812 women, 967 men). The baseline examination took place between December 2002 and January 2006; the response was 64.1% (68.6% for men, and 59.5% for women; see details in [17]).

Data collection included a detailed standardized, computer-assisted interview, questionnaires and a physical examination by a trained study nurse. Details of the study design have been described elsewhere [17,18]. The CARLA Study was approved by the ethics committee of the Medical Faculty of the Martin-Luther-University Halle-Wittenberg and by the State Data Privacy Commissioner of Saxony-Anhalt; written informed consent was obtained from all respondents.

### Assessment of socioeconomic indicators

During the interview, sociodemographic variables were collected, including information on school education and vocational training, household income, number of household members, and occupational position in the current or - if not working - in the last occupation. Assessment of education, occupation and income was based on German guidelines [19,20].

Education was constructed as a three-level combination of school and vocational education, the lowest category indicating low to medium school qualification without a completed vocational qualification, the highest category a school degree with university qualification and/or a completed university degree. Occupational position was defined as either low (blue-collar worker, farmer), middle (foremen, qualified employee, self-employed with <10 employees), or high (highly qualified employee, supervisor, self-employed with ≥10 employees or in academic professions). We calculated income as the monthly equivalent household net income according to the OECD-modified scale [21] which takes into account the number of household members. The respective categories were <750 € (low), 750-<2000 € (middle), and ≥2000 € (high). Childhood SES was defined by parental SES during the childhood of the subject. Parental education reflects the cultural resources available to the child, and occupation of parents is related both to the family's position in the social hierarchy and to its economic resources. For each parent of the respondent, an SES score was calculated from combined information on school education and occupational position. The final three-level aggregated index 'childhood socioeconomic status' was derived from the respective SES scores of both parents, the father being accorded double weighting. If one parent was missing, only the information of the other parent was used. A low childhood SES indicated a mainly working-class background and low education (<10 years), whereas respondents with a medium childhood SES had mainly white-collar parents with a medium school education. A high childhood SES indicated high parental education in combination with self-employment or white-collar employment.

### Cardiovascular risk factors

The medical examination included among others anthropometric and blood pressure measurements. For our analyses, we used the average of the second and third of three measurements of sitting systolic blood pressure (automated oscillometric measurement device) in our analyses. Hypertension was defined as SBP >= 140 and/or DBP >= 90 mmHg, or use of antihypertensive medication (self-reported or by ATC code).

We chose waist-hip-ratio (WHR) to define abdominal obesity because of its high predictive value for coronary heart disease [15,22,23]. Waist and hip circumference were measured following standard procedures also applied in the MONICA/KORA and SHIP studies [24,25]. Abdominal obesity was defined as a WHR of >1.0 for men and of >0.85 for women, respectively [22,26]. Body mass index (BMI) was calculated as kg/m^2^.

Current smoking status was used as a behavioural risk factor for cardiovascular disease. All respondents who were currently smoking at least once a week one cigarette, pipe or cigar over at least one year were defined as smokers.

### Statistical methods

All analyses were conducted with SAS 9.2 (SAS Institute, Cary, NC, USA), for men and women separately. We calculated logistic and linear regression models to analyse the influence of each SES indicator (childhood SES, education, occupational position and income) on cardiovascular risk factors (systolic blood pressure, abdominal obesity, smoking). Age-adjusted models were calculated for all four SES indicators separately (model 1). The fully adjusted model contained all four SES indicators simultaneously (model 2). For systolic blood pressure, linear regression was conducted with the SAS procedure 'proc mixed', and adjusted means were calculated with the statement 'lsmeans' for each SES category (low, middle, high). For the binary outcomes smoking and abdominal obesity, the SAS procedure 'proc logistic' was applied. For all SES indicators, the highest category was set as the reference group. 95% confidence intervals were given.

Results were evaluated in terms of both statistical significance and effect size, the latter indicating findings which might be clinically important and require further investigation. Interpretation of results was thus based on the statistical significance of effects at α = 0.05 as well as on the clinical relevance of effect size. An association was assumed as clinically relevant if (for binary outcomes) the respective age-adjusted odds ratio (model 1) was >1.5 or ≤0.67, or (for systolic blood pressure) age-adjusted mean differences to the reference category were ≥5 mmHg. We tested whether the whole complex of four SES indicators was statistically significant by comparing the full model (including age, education, occupation, income and childhood SES) with a simple model containing age as the only independent factor. The difference of model fit (-2 log likelihood values) was calculated, and its significance (chi2-test) determined at α = 0.05.

The hypothesized age or cohort effect in the association between education and CVD risk factors was tested by introducing an interaction term of age and education in the regression models at α = 0.10. The respective estimated means of systolic blood pressure and odds ratios for binary outcomes at four different age points were displayed graphically.

## Results

### Study population

The characteristics of the study population are given in table 1.

**Table 1:** Characteristics of the study population (CARLA baseline study)

	Men	Women
N, %	976 (54.4)	812 (45.6)

Age, mean (SD)	64.4 (10.2)	63.3 (10.0)

**Socioeconomic factors**		

Education, N (%)		

Low	36 (3.7)	121 (14.9)

Middle	592 (61.2)	536 (66.0)

High	339 (35.1)	155 (19.1)

Occupational position, N (%)		

Low	247 (25.6)	153 (19.0)

Middle	372 (38.6)	533 (66.1)

High	346 (35.9)	120 (14.9)

Income, N (%)		

Low	95 (9.9)	117 (14.6)

Middle	778 (81.2)	644 (80.6)

High	85 (8.9)	38 (4.8)

Childhood SES, N (%)		

Low	523 (55.1)	457 (57.2)

Middle	375 (39.5)	299 (37.4)

High	52 (5.5)	43 (5.4)

**Cardiovascular risk factors and diseases**		

Systolic blood pressure, mean (SD)	145.9 (19.8)	141.7 (22.8)

Diastolic blood pressure, mean (SD)	85.9 (11.1)	83.2 (10.9)

Hypertension, N (%)	794 (82.1)	611 (75.3)

Body mass index, mean (SD)	28.2 (4.1)	28.5 (5.4)

Waist-hip-ratio, mean (SD)	1.00 (0.06)	0.88 (0.06)

Abdominal obesity, N (%)	507 (52.4)	604 (74.4)

Current smoker, N (%)	222 (23.0)	117 (14.4)

CHD, N (%)	121 (12.5)	25 (3.1)

CVD, N (%)	153 (15.8)	48 (5.9)

SES, socioeconomic status. SD, standard deviation. Hypertension: blood pressure >140/90 mmHg or current use of antihypertensives. Abdominal obesity: waist-hip-ratio >1.0 (men)/>0.85 (women). CHD, coronary heart disease (myocardial infarction, coronary artery bypass surgery, percutaneous transluminal coronary angioplasty). CVD, cardiovascular disease (CHD, stroke, carotid surgery)

The mean age of participants was 64.4 years for men and 63.3 years for women. The majority had a middle or high educational background, the frequency of low education being higher in women than in men (14.9% vs. 3.7%, respectively). About one-third of men, but only 15% of women had a high occupational position; more than 80% of participants reported a middle income. More than half had a low childhood socioeconomic status, indicating the predominance of working-class background in this population. SES indicators were moderately correlated, with Spearman's correlation coefficients ranging from 0.12 (for childhood SES and income in women) to 0.62 (for education and occupation in men). Correlations of the majority of SES indicators were higher in men than in women (data not shown).

In general, we found a high prevalence of cardiovascular risk factors in this population. The mean systolic blood pressure was 145.9 mmHg in men, and 141.7 mmHg in women; prevalence of hypertension (defined as BP >140/90 mmHg or use of antihypertensive drugs) was over 80% in men and 75% in women. Whereas prevalence of abdominal obesity, mean BMI and WHR were rather high, less than a quarter of men and 14% of women were currently smokers.

### Association of SES and cardiovascular risk factors

Results of multivariate linear and logistic regression models are displayed in table 2.

**Table 2:** Cardiovascular risk factors by socioeconomic factors - adjusted means and odds ratios with 95% confidence intervals

	Systolic blood pressure (mean, mmHg)		Abdominal obesity (OR)		Smoking (OR)	
	**Age-adjusted**	**Fully adjusted^1^**	**Age-adjusted**	**Fully adjusted^1^**	**Age-adjusted**	**Fully adjusted^1^**

**MEN**						

**Education**						

Low	142.0 (135.5; 148.4)	142.9 (135.4; 150.5)	1.08 (0.54; 2.15)	1.05 (0.46; 2.39)	3.44 (1.58; 7.51)	2.41 (0.93; 6.25)

Middle	146.1 (144.5; 147.7)	145.6 (142.5; 148.6)	1.52 (1.16; 1.99)	1.39 (0.97; 1.98)	1.79 (1.25; 2.55)	1.40 (0.88; 2.24)

High	145.9 (143.8; 148.0)	146.1 (142.7; 149.5)	1.00	1.00	1.00	1.00

p-value/relevance^2^	0.470	0.746	**0.008**	0.160	**0.001**	0.158

p-value of interaction education with age	0.402	-	0.785	-	0.125	-

**Occupational position**						

Low	146.9 (144.4; 149.4)	146.8 (142.6; 150.9)	1.35 (0.97; 1.89)	0.96 (0.61; 1.50)	1.92 (1.26; 2.90)	1.11 (0.63; 1.97)

Middle	145.7 (143.7; 147.7)	144.4 (140.5; 148.3)	1.49 (1.11; 2.00)	1.18 (0.82; 1.69)	1.49 (1.00; 2.20)	1.10 (0.68; 1.77)

High	145.3 (143.2; 147.4)	143.4 (139.4; 147.4)	1.00	1.00	1.00	1.00

p-value/relevance^2^	0.625	0.281	0.028	0.417	**0.009**	0.917

**Income**						

Low	143.7 (139.6; 147.8)	142.8 (137.8; 147.7)	1.89 (1.04; 3.43)	1.63 (0.85; 3.14)	3.59 (1.83; 7.06)	2.29 (1.08; 4.86)

Middle	146.0 (144.6; 147.4)	144.9 (141.7; 148.0)	1.81 (1.13; 2.90)	1.57 (0.95; 2.58)	1.41 (0.80; 2.49)	1.07 (0.58; 1.98)

High	147.6 (143.4; 151.9)	146.9 (141.6; 152.3)	1.00	1.00	1.00	1.00

p-value/relevance^2^	0.409	0.436	**0.040**	0.202	**<.001**	0.011

**Childhood SES**						

Low	145.7 (144.0; 147.4)	145.1 (141.9; 148.2)	1.43 (0.80; 2.55)	1.12 (0.61; 2.06)	1.76 (0.83; 3.73)	1.16 (0.53; 2.56)

Middle	146.6 (144.6; 148.6)	146.2 (142.8; 149.6)	1.25 (0.69; 2.24)	1.06 (0.58; 1.94)	1.60 (0.75; 3.42)	1.23 (0.56; 2.67)

High	143.7 (138.4; 149.1)	143.3 (137.1; 149.5)	1.00	1.00	1.00	1.00

p-value/relevance^2^	0.580	0.527	0.351	0.897	**0.319**	0.860

**p-value of full SES complex**	-	0.736	-	0.041	-	0.003

						

**WOMEN**						

**Education**						

Low	146.0 (142.0; 150.0)	143.2 (137.6; 148.8)	1.45 (0.81; 2.60)	0.89 (0.42; 1.86)	2.69 (1.30; 5.60)	1.41 (0.53; 3.74)

Middle	141.3 (139.4; 143.1)	137.8 (133.7; 141.9)	1.43 (0.96; 2.12)	1.01 (0.62; 1.65)	1.38 (0.80; 2.36)	1.13 (0.58; 2.23)

High	139.9 (136.3; 143.4)	137.6 (133.1; 142.1)	1.00	1.00	1.00	1.00

p-value/relevance^2^	**0.064**	0.103	0.192	0.901	**0.027**	0.775

p-value of interaction education with age	0.173	-	0.040	-	0.044	-

**Occupational position**						

Low	141.6 (138.1; 145.1)	138.2 (133.2; 143.1)	1.73 (1.01; 2.99)	1.42 (0.72; 2.81)	2.41 (1.22; 4.74)	1.72 (0.71; 4.10)

Middle	142.3 (140.5; 144.2)	140.6 (136.8; 144.4)	1.51 (0.98; 2.33)	1.27 (0.76; 2.11)	1.08 (0.59; 2.00)	0.88 (0.43; 1.82)

High	139.7 (135.7; 143.7)	139.9 (134.7; 145.1)	1.00	1.00	1.00	1.00

p-value/relevance^2^	0.496	0.550	**0.099**	0.576	**0.003**	0.066

**Income**						

Low	143.4 (139.4; 147.5)	140.4 (135.6; 145.1)	2.80 (1.25; 6.23)	2.12 (0.90; 4.97)	5.78 (1.86; 18.03)	5.41 (1.62; 18.08)

Middle	141.6 (139.9; 143.4)	140.1 (137.2; 143.0)	1.58 (0.80; 3.14)	1.34 (0.65; 2.73)	2.30 (0.78; 6.82)	2.16 (0.70; 6.67)

High	138.8 (131.7; 146.0)	138.2 (130.6; 145.7)	1.00	1.00	1.00	1.00

p-value/relevance^2^	0.507	0.870	**0.025**	0.149	**<0.001**	0.001

**Childhood SES**						

Low	143.1 (141.1; 145.1)	143.3 (140.0; 146.6)	2.27 (1.18; 4.38)	1.94 (0.95; 3.95)	0.79 (0.34; 1.84)	0.50 (0.20; 1.29)

Middle	140.6 (138.1; 143.1)	140.7 (136.9; 144.4)	1.76 (0.90; 3.44)	1.58 (0.78; 3.22)	0.85 (0.36; 2.03)	0.67 (0.26; 1.72)

High	134.2 (127.7; 140.8)	134.7 (127.5; 141.9)	1.00	1.00	1.00	1.00

p-value/relevance^2^	**0.020**	0.042	**0.031**	0.148	0.841	0.248

**p-value of full SES complex**	-	0.077	-	0.094	-	< 0.001

^1 ^adjusted for age and all other SES indicators

^2 ^p-value: F-test (systolic blood pressure) or Chi^2^-test (abdominal obesity, smoking). Relevance: bold p-values in age-adjusted models indicate relevant differences of at least one category to the respective high SES category (>= 5 mmHg for systolic blood pressure, OR >= 1.5 or <= 0.67 for abdominal obesity and smoking).

OR, Odds Ratio. SES, Socioeconomic status

In men, none of the SES indicators was related to systolic blood pressure in terms of statistical significance or relevance of effect size - differences in mean values were small between respective SES categories. No statistically significant association was observed between childhood SES and systolic blood pressure, abdominal obesity and smoking. We found statistically significant relationships of education, occupational position and income with abdominal obesity and smoking in men. The majority of these associations was also clinically relevant in terms of effect size with odds ratios >= 1.5. For obesity, a social gradient was seen only in income whereas with respect to education, the middle category displayed the highest risk (OR 1.52, 95% CI 1.16-1.99). The risk of being a current smoker decreased with increasing education, occupation and income. Men with low income had a more than threefold risk of smoking than men with high income (OR 3.59, 95% CI 1.89-7.06). The association between income and smoking remained statistically significant after adjustment for the three other SES indicators education, occupational position, and childhood SES (p = 0.01).

For women, in age-adjusted models childhood SES was statistically significantly associated with systolic blood pressure and abdominal obesity. These associations were also clinically relevant in terms of effect size, with blood pressure differences of >5 mmHg and odds ratios of >1.5 for obesity compared with the highest SES category. Mean blood pressure of women with a low childhood socioeconomic status was about 9 mmHg higher than that of women with a high SES. The three adult SES indicators education, occupational position and income were significantly and relevantly related to smoking, and income also to abdominal obesity. Women with low income had a nearly sixfold risk of smoking compared with women with high income (OR 5.78, 95% CI 1.86-18.03). Adjustment for all other SES indicators attenuated the influence of income only to a small degree (OR 5.41, CI 1.62-18.08). A clinically relevant blood pressure difference of approximately 6 mmHg was observed between women of low and high education; the association approached statistical significance (p = 0.06).

The influence of the whole complex of four SES indicators on smoking was statistically significant both in men and in women (p-values <0.05), and on abdominal obesity in men only. No significant influence on systolic blood pressure was observed.

### Interaction of age and education

**Figure 1:**
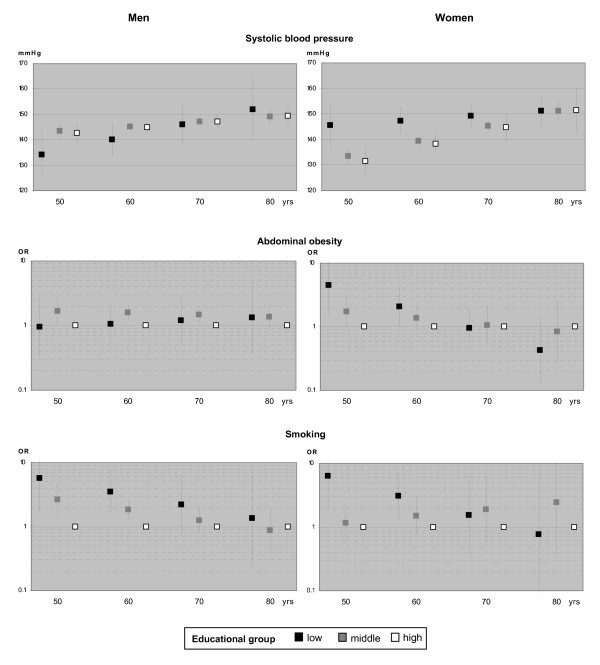
**Interaction of education and age on cardiovascular risk factors - adjusted means and odds ratios with 95% confidence intervals by educational group at four age points**.

In men, age did not statistically significantly modify the effect of education on any of the outcomes. In women, we found statistically significant interaction effects between education and age for abdominal obesity as well as for smoking. Figure 1 displays age-adjusted means and odds ratios for the respective outcomes by educational groups at the age points of 50, 60, 70 and 80 years.

In men, for systolic blood pressure and abdominal obesity, at all ages only small differences between educational groups were observed. For smoking, the social gradient decreased with age as we had hypothesized, although statistically non-significantly. In women, there was an age-dependent social gradient for all three outcomes, with group differences decreasing at higher ages. At the age of 70, the gradient in abdominal obesity had levelled off and at 80 had even reversed to some degree. In smoking, on the other hand, the negative impact of middle education compared with high education increased with age, whereas for women in the lowest educational group it decreased. Since prevalence of smoking in older subjects was low, however, effect estimates were rather imprecise with large confidence intervals.

## Discussion

The aim of this study was to investigate the role of different socioeconomic indicators for cardiovascular risk factors in a middle to old aged population. Associations were assessed in terms of both statistical significance and clinical relevance of effect size. Indicators of adult socioeconomic status (education, occupational position and income) were influential, particularly on lifestyle-related CVD-risk factors (smoking, obesity) in respect of both statistical significance and clinical relevance of effect size. As regards systolic blood pressure, only for female education and childhood SES we found a clinically relevant although statistically non-significant association.

The most relevant risk factor for smoking in terms of effect size was low income, particularly in women. In the majority of statistically significant associations, there was a clear social gradient indicating increasing risk with decreasing SES. Even in non-significant associations, the respective highest SES category nearly always had the lowest risk.

Although socioeconomic status is an established risk factor for health outcomes and health behaviour, there are also studies showing varying patterns depending on gender, SES indicator and outcome investigated. In an analysis of the NHANES III Study, Winkleby [3] found a significant association of education with hypertension in women, but not in men, whereas income was unrelated to hypertension in both sexes. Several other studies confirm the lack of association between SES and blood pressure in men [7,27].

In a systematic review of SES and obesity including over 300 studies, McLaren [6] concluded that the social gradient found in women was frequently absent in men, for whom many results were non-significant or indicated a reversed gradient. In the CARLA Study, male participants with middle education, occupational grade or income had the same or even higher risk of being obese than men of the lowest category. According to McLaren [6], this might be attributed to the fact that for men more so than for women a larger body size is valued as a sign of physical dominance, leading to contradictory influences on lifestyle-related behaviour such as diet and physical activity. Many studies, however, use only binary indicators of social status, which might disguise differences between lower and middle class, and only one indicator of obesity. Silventoinen et al. [28], on the other hand, found significant associations between education and obesity defined as abdominal obesity (both as binary and continuous variable) and defined as high body mass index.

As regards smoking, Laaksonen et al. [8] reported generally larger SES effects in women than in men. Income was related to smoking for both sexes, whereas for education and occupational position, the association was statistically significant only in women. In an analysis of the British Women's Heart and Health Study, education, occupation and paternal occupation were related to ever having smoked [29]. Comparison with our results, however, is hampered by the fact that different definitions of socioeconomic status and smoking status were used.

In our study, childhood SES had a relevant and statistically significant influence on cardiovascular risk factors in women (systolic blood pressure, abdominal obesity). In men, on the other hand, we found a relevant but statistically non-significant association with smoking. This is in agreement with a systematic review by Senese et al. [30], who in the majority of included studies found significant associations between childhood socioeconomic factors and adult obesity in women, but not in men. Power et al. [31] showed that paternal occupational position was related to blood pressure and body mass index at the age of 45; after adjustment for adult SES, most associations were attenuated to some degree. In an international comparison, the majority of studies reported negative associations of childhood SES and current smoking in women, whereas in men no consistent pattern was found [32]. This is contrary to our findings where for men, there was a relevant although statistically non-significant increase in risk with lower childhood SES, and women of lower social origin had a somewhat lower risk of smoking than women of high childhood class.

The literature on life course epidemiology has increased considerably during the last years [33-35]. According to Kuh et al. [35], the link between childhood factors and adult health might be via adverse childhood exposures such as poor diet, or via learning experiences and social capital that affect adult behavioural and socioeconomic factors. In our female population, the influence of childhood SES on blood pressure and obesity was attenuated only slightly when adult SES was taken into account. This supports the assumption of long-term biological processes which are to some extent independent of adult social factors. Further analyses need to investigate gender differences in these pathways.

Little is known so far about age or cohort effects in social inequalities. According to our results, the influence of education on cardiovascular risk factors in men is not statistically significantly modified by age, whereas in women there was a significant interaction of age and education on risk of obesity and smoking. In general, however, differences between educational groups tended to decrease with higher age both in men and in women. Galobardes et al. [10] point out that owing to the social gradient in mortality, elderly people with a low socioeconomic status might be a selected group (who survived despite adverse living conditions), resulting in the narrowing of health inequalities in the elderly. This explanation would indicate an age effect in selective survival rather than at a birth cohort effect caused by the changing evaluation of educational qualifications, as we hypothesized. Decreasing health inequalities might also support the age-as-leveller hypothesis of Dupre [9] which states that the negative effect of social disadvantage decreases during lifetime. In our cross-sectional design, however, a differentiation between birth cohort and age effects is not possible.

### Strengths and limitations

Our study has the advantage of a representative population-based sample; social indicators and cardiovascular risk factors were assessed in a highly standardized way in agreement with other German and international studies. On the other hand, it has the usual limitations of a cross-sectional design where independent variables and outcomes are measured at the same time. Non-responders of the CARLA Study were less educated and showed partly more adverse health behaviours. We thus cannot exclude a selection bias (inclusion of subjects with higher SES and better risk profiles), which might shift the observed effect estimates towards the null, as compared with the true underlying associations of SES with the respective cardiovascular risk factors (see [18] for a non-responder analysis).

Recall bias might have an impact on quantification, particularly of those social factors that had been acquired many years ago. Our categories of education, occupational position and income were rather broad, however, and therefore we assume that misclassification is negligible. For childhood socioeconomic status, we used an aggregated index which might disguise the impact of single components such as paternal occupation on health.

The outcomes that were investigated included both continuous (systolic blood pressure) and dichotomous variables (abdominal obesity, smoking). This hampers comparison of the strength of association between SES indicators and different CVD risk factors. Some subgroups of social indicators and smoking were small, especially in higher age groups, leading to imprecise estimates with large confidence intervals. A merging of two social categories (e.g. low and middle education) would increase power but also blur relevant and remarkable group differences. Our dual evaluation of results in terms of statistical significance and clinical relevance of effect size, although unusual in epidemiological research, was intended to highlight associations between social status and cardiovascular risk factors that might, owing to small sample size, be clinically relevant though statistically non-significant. One must, however, bear in mind that these observed large effect sizes may still be owed to chance. Interaction of education and age was tested on a multiplicative rather than an additive scale. This approach, although highlighting the influence of education on CVD risk factors at different ages, does not quantify potential differences in excess risk by educational group.

## Conclusions

We conclude that even socioeconomic attributes that have been acquired a long time ago (childhood socioeconomic status, education) can have an impact on lifestyle-related CVD risk factors in the elderly. As regards a social gradient, in our study the association of social status with these risk factors appears more consistent for women than for men.

## Abbreviations

BMI: Body mass index; CARLA: Cardiovascular disease, living and ageing in Halle Study; CHD: Coronary heart disease; CI: Confidence interval; CVD: Cardiovascular disease; DFG: German Research Foundation (Deutsche Forschungsgemeinschaft); KORA: Cooperative Health Research in the Augsburg Region; MONICA: Monitoring Trends and Determinants in Cardiovascular Disease; NHANES III: Third National Health and Nutrition Examination Survey; OECD: Organisation for Economic Co-operation and Development; OR: Odds ratio; SD: Standard deviation; SES: Socioeconomic status; SHIP: Study of Health in Pomerania; WHR: Waist-hip-ratio

## Competing interests

The authors declare that they have no competing interests.

## Authors' contributions

BS helped to coordinate the CARLA Study, conducted the SES analyses and wrote the manuscript. AK helped to coordinate the CARLA Study and draft the manuscript. DT helped to coordinate the CARLA Study and draft the manuscript. KW helped to design the CARLA Study and draft the manuscript. JH helped to design the CARLA Study, conduct the statistical analyses and draft the manuscript. KHG designed major parts of the CARLA Study, coordinated the study, and helped to draft the manuscript. All authors have read and approved the final version of the manuscript.

## Pre-publication history

The pre-publication history for this paper can be accessed here:

http://www.biomedcentral.com/1471-2458/11/289/prepub
